# QTL affecting stress response to crowding in a rainbow trout broodstock population

**DOI:** 10.1186/1471-2156-13-97

**Published:** 2012-11-07

**Authors:** Caird E Rexroad, Roger L Vallejo, Sixin Liu, Yniv Palti, Gregory M Weber

**Affiliations:** 1 USDA/ARS National Center for Cool and Cold Water Aquaculture, Leetown, WV, USA

## Abstract

**Background:**

Genomic analyses have the potential to impact selective breeding programs by identifying markers that serve as proxies for traits which are expensive or difficult to measure. Also, identifying genes affecting traits of interest enhances our understanding of their underlying biochemical pathways. To this end we conducted genome scans of seven rainbow trout families from a single broodstock population to identify quantitative trait loci (QTL) having an effect on stress response to crowding as measured by plasma cortisol concentration. Our goal was to estimate the number of major genes having large effects on this trait in our broodstock population through the identification of QTL.

**Results:**

A genome scan including 380 microsatellite markers representing 29 chromosomes resulted in the *de novo* construction of genetic maps which were in good agreement with the NCCCWA genetic map. Unique sets of QTL were detected for two traits which were defined after observing a low correlation between repeated measurements of plasma cortisol concentration in response to stress. A highly significant QTL was detected in three independent analyses on Omy16, many additional suggestive and significant QTL were also identified. With linkage-based methods of QTL analysis such as half-sib regression interval mapping and a variance component method, we determined that the significant and suggestive QTL explain about 40-43% and 13-27% of the phenotypic trait variation, respectively.

**Conclusions:**

The cortisol response to crowding stress is a complex trait controlled in a sub-sample of our broodstock population by multiple QTL on at least 8 chromosomes. These QTL are largely different from others previously identified for a similar trait, documenting that population specific genetic variants independently affect cortisol response in ways that may result in different impacts on growth. Also, mapping QTL for multiple traits associated with stress response detected trait specific QTL which indicate the significance of the first plasma cortisol measurement in defining the trait. Fine mapping these QTL can lead towards the identification of genes affecting stress response and may influence approaches to selection for this economically important stress response trait.

## Background

Employing molecular approaches to understanding complex genetic traits which are difficult or expensive to measure has the potential to facilitate genetic improvement through selective breeding and the identification of population specific genetic variants which affect important phenotypes [1]. Although some populations may exhibit similar phenotypes, the genetic architectures underlying a trait may be vastly different. Such traits include those associated with the physiological responses of rainbow trout to the stressors of aquaculture production environments. Typical stressors can be categorized under handling and manipulation, overcrowding, sub-optimal water quality parameters, and social interactions, all of which have been shown to negatively affect production traits such as growth, feed intake, feed efficiency, disease resistance, flesh quality, and reproductive performance [2-7]. Fish respond to these stressors in similar patterns to those exhibited by terrestrial vertebrates, by stimulating the hypothalamic-sympathetic-chromaffin cell axis which releases catecholamines to increase oxygen uptake and energy mobilization and the hypothalamic-pituitary-interrenal axis, which produces cortisol to effect carbohydrate, protein, and lipid metabolism [8]. As in mammals, an appropriate hormonal response enables the fish to endure a stress; however, excessive or prolonged exposure to these hormones, particularly cortisol, can also have deleterious effects on traits associated with aquaculture production [3].

Despite clear evidence that these hormonal responses are central to the overall stress response, attempts to use measures of the hormonal responses as predictors of performance in rainbow trout, have been equivocal at best. A good example is the response to crowding stress in rainbow trout. In an European population, response to crowding stress was observed to be heritable, with fish responding with elevated plasma cortisol concentrations having inferior growth when reared in co-culture with low responders [9,10]. Similarly studies on fish in the US have also shown that this trait is heritable; however, in contrast, fish responding to stress with elevated concentrations of plasma cortisol were observed to be superior growers [11,12]. These differences in association between the physiological marker for stress response and the production trait indicates genetic variation in multiple biochemical mechanisms controlling growth and/or stress responses in these populations. Therefore our goal was to survey multiple families from a single broodstock population to characterize the diversity of loci potentially affecting stress response to crowding as a first step in using allelic variation for parsing out the crude phenotype of post-stressor plasma cortisol levels into more refined phenotypes that will likely better associate with production traits as predicted.

In 2002 the National Center for Cool and Cold Water Aquaculture (NCCCWA) initiated a selective breeding program with genetically diverse strains of rainbow trout with the aim of creating stocks that are improved for aquaculture production efficiency [13]. To date selection has focused on characteristics associated with growth and disease resistance [14-16]. Stress has been shown to affect both of these traits [8]. For initial evaluation seven multi-generation families from the growth line were selected for identifying quantitative trait loci (QTL) for stress response based on their plasma cortisol response to crowding [12]. Previously, complex segregation analysis (CSA) has suggested that one or more major genes is affecting stress response to crowding in this population, and that a dominant gene has a large negative effect on plasma cortisol concentration in response to crowding stress [17]. Therefore our goals in this study were to: 1) identify the number of loci affecting stress response in our broodstock population; and 2) validate the findings of the previous CSA. To this end we evaluated seven families containing a total of 222 offspring that were measured for their stress response according to the protocol of Pottinger and Carrick [18] as modified by Weber and Silverstein [12], and conducted a genome scan with 380 microsatellite markers selected from the NCCCWA genetic map [19]. We defined two traits for QTL mapping. One phenotype was based on estimated breeding values (EBV) using four plasma cortisol repeated measurements per animal. The second phenotype was based on best linear unbiased predictors of the last three repeated measurements (BLUP3) as we observed that the initial cortisol measurement for each fish did not correlate well with the last three measurements.

## Methods

The stress challenge and collection of blood for measuring plasma cortisol concentration was conducted with the approval of the IUCAC of the USDA/ARS National Center for Cool and Cold Water Aquaculture, protocol #50.

### Mapping population

The QTL mapping population was identified from a broodstock population at the NCCCWA in Leetown, West Virginia, USA. This selective breeding program was initiated in 2002 and designed to select for growth in even year classes and disease resistance in odd year classes [13]. Stress responsiveness was initially evaluated to determine whether or not it is associated with performance traits affecting aquaculture production efficiency. To this end a total of 584 fish representing 64 families and 9 replicated families (8 fish per family) from the 2002 year class were evaluated for stress response to crowding according to the protocol of Pottinger and Carrick [9] to identify phenotypic variation of 11.6-93.9 ng cortisol/mL and a significant positive association between plasma cortisol response and growth performance [12]. In the 2004 year class, 8 fish from each of the top 15% of the growth selected families were similarly evaluated for stress response to identify a heritability of 46%; mean family values were used to develop a high and low responding (P_1_) generation [20]. In 2006, high and low responding P_1_ fish were crossed to create a F1 generation consisting of 7 full-sib (FS) families including 222 offspring from 12 P_1_ parents which were evaluated for their potential for use in understanding the genetic control of this trait [21].

### Stress challenge

The stress challenge method for crowding was modified from Pottinger and Carrick [9] as described by Weber and Silverstein [12]. The challenge for the parental generation is described in Weber *et al.*[20] and for the offspring in Vallejo *et al.*[17] Briefly, fish were reared as individual families and were exposed to an artificial ambient photoperiod and reared in continuous flow spring water with temperatures between ~11.5 and 13.5°C and dissolved oxygen near saturation. Fish were placed in 120 l blue polypropylene tanks at approximately one month of age. Seven weeks prior to the start of the experiment, fish were tagged with passive integrated transponder (PIT) tags (Avid Identification Systems Inc., Norco CA) and split by family into tanks containing eight fish each. Fish in the stress study were fed Zeigler Gold (Zeigler Bros. Inc., Gardners PA) at 2% body weight/day. The eight fish from each tank were sampled four times at 4-week intervals when the fish were approximately 160 g body weight. Fish were not fed the day of sampling or the afternoon preceding the sampling. For the crowding stress challenge, fish from a single family were netted and transferred from a 120 l tank to a 6 l or 15 l tank and left undisturbed for three hours. The 6 l or 15 l tanks were used in an effort to keep fish densities in the challenge consistent as fish continue to grow throughout the experiment. After three hours of crowding, the fish were then netted and transferred into an anesthesia bath followed by blood collection. Plasma cortisol was measured (ng/ml) by tritium radioimmunoassay following procedures described by Redding *et al.*[22].

### Stress response derived traits

The parents and offspring fish used in this study (sires = 5; dams = 7; offspring = 222) had four repeated measurements of plasma cortisol recorded at about 4-week intervals as described elsewhere [17]. These plasma cortisol measurements are highly variable and heavily impacted by environmental effects with a polygenic model heritability of ~0.26 [17]. So, in order to minimize the plasma cortisol variation due to non-genetic factors, and consequently increase the statistical power of QTL detection, and reduce the false positive rate of detected QTL, we derived two stress response phenotypes to use in QTL analyses.

First, we estimated animal breeding value (EBV) using four repeated measurements of plasma cortisol and fitting a mixed inheritance linear model under a Bayesian framework with software iBay version 1.46 [23]. We decided to fit a permanent environmental effect in the repeated measures mixed model analysis to account for the covariance between the records of an individual (i.e., repeated measurements), and capture individual variation across measurements in the EBV computation. The covariates body weight, body length and sexual maturity which had significant effect on the predictive power of the response variable plasma cortisol [17] were also included in the mixed model to minimize the variance in the sampled population.

Second, we also estimated an animal effect using best linear unbiased prediction (BLUP3) and fitting three repeated measurements of plasma cortisol in a multivariate mixed model with the software ASReml version 2.0 [24]. The mixed model also included covariates indicated above that had significant effect on the predictive power of the response variable plasma cortisol. Here, we estimated heritability of each repeated measurement and genetic correlations between repeated measurements of plasma cortisol with ASReml version 2.0 [24], and noticed that measurement at time 1 was different than measurements at time 2, 3 and 4 in heritability and genetic correlations. So, we decided to calculate an index BLUP3 that was weighted by their relative heritability using measurements at time points 2, 3 and 4, respectively.

In order to determine the effect of using adjusted animal effects for mid-parent genetic effect in the QTL analysis, we performed QTL analysis using HS regression interval mapping as outlined above using offspring animal effects that were adjusted for mid-parent genetic effects. The adjustment of offspring animal effects for mid-parent genetic effect allowed accounting for the effects of relatives, and the use of a pure measure of offspring individual’s genetic value in the QTL analysis [25,26].

As expected, we determined that the estimated animal effects EBV and BLUP3 had a Pearson’s correlation of *r* = 0.84 (*P* <0.0001) with SAS Procedure REG [27]. In this study, we used the estimated animal effects EBV and BLUP3 in the QTL analysis. The use of estimated animal effects such as EBV and daughter-yield deviation (DYD) in whole genome QTL scans has been well documented in livestock species [25,28-31].

### Genotyping and linkage analysis

A panel of 412 microsatellite loci identified from the NCCCWA Genetic Map [19] were used to genotype the parents and offspring of seven FS QTL mapping families (sires = 5; dams = 7; offspring = 222; total marker genotyped fish = 234). Markers were either genotyped using the tailed protocol [32] or by direct fluorescent labelling (with FAM, HEX, or NED) of the forward primer. Primer pairs were obtained from commercial sources (forward primers labelled with FAM or HEX from Alpha DNA, Montreal, Quebec, Canada, or NED from ABI, Foster City, CA, USA). PCR reactions consisted of 12 μl reaction volumes containing 12.5 ng DNA, 1.5-2.5 mM MgCl_2_, 1.0 μM of each primer, 200 μM of dNTPs, 1X manufacturer’s reaction buffer and 0.5 units Taq DNA polymerase. Thermal cycling consisted of an initial denaturation at 95°C for 15 min followed by 30 cycles of 95°C for 1 min, annealing temperature for 45 s, 72°C extension for 45 s and a final extension at 72°C for 10 min. PCR products were visualized on agarose gels after staining with ethidium bromide. Markers were grouped in combinations of two or three markers based on differences in fluorescent dye color and amplicon size. Three μl of each PCR product was diluted with 20 μl of water, 1 μl of the diluted sample was added to 12.5 μl of loading mixture made up with 12 μl of HiDi formamide and 0.5 of Genscan 400 ROX internal size standard. Samples were denatured at 95°C for 5 min and kept on ice until loading on an automated DNA sequencer ABI 3730 DNA Analyzer (ABI, Foster City, CA, USA). Output files were analyzed using GeneMapper version 3.7 (ABI, Foster City, CA, USA), formatted using Microsoft Excel and stored in Microsoft Access database. Linkage maps were constructed for each chromosome using marker genotypes from all seven mapping families with the software MULTIMAP version 2.0 [33] using the approach of Rexroad *et al*[19].

### Testing loci for Mendelian segregation distortion

Before QTL analysis, all STR loci were tested for Mendelian segregation distortion (MSD). In outbreed populations, the progeny of an informative QTL mapping family can have any of these marker genotype proportions: 1:1; 1:2:1 and 1:1:1:1. Within individual FS families, the marker genotype counts were performed using a Perl script (Written by G. GAO, unpublished). Then, Chi-Square goodness-of-fit test of marker genotype counts to expected proportions under Mendelian segregation was performed with SAS Procedure FREQ (SAS, 2007) using a default significance level of α = 0.01. Loci with significant MSD were not used in the QTL analysis unless these loci had high quality marker genotypes (i.e., minimum genotyping errors). The rationale to use STR loci that had significant MSD in QTL genome scan is that loci with significant MSD can be linked to viability/survivability and stress response QTL.

### QTL analysis using HS regression interval mapping

For each stress response trait, we performed combined sire-family (five HS families) and dam-family (seven HS families) HS regression analysis, separately, using the web-based software GridQTL [34]. This software implements a multi-marker approach of interval mapping in HS families as described by Knott *et al.*[35]. This method of QTL analysis does not assume the parents had fixed QTL alleles, instead it relaxes the assumption of fixed QTL allele [35]. A QTL with a gene substitution effect is fitted at 1-cM intervals along the chromosome using this one-QTL model, *y*_*ij*_ = *a*_*i*_ + *b*_*i*_*x*_*ij*_ + *e*_*ij*_ where *y*_*ij*_ is trait score of individual *j* from sire or dam *i; a*_*i*_ is average effect for HS family *i; b*_*i*_ is regression coefficient within HS family *i* (substitution effect of QTL); *x*_*ij*_ is conditional probability for individual *j* within HS family *i* of inheriting allele 1 (or 2); and *e*_*ij*_ is the residual error.

In the HS regression analysis, the likelihood ratio (LR) test statistic was defined as $LR=-2\ln\left[\frac{{\hat{l}}_{r}\left(z\right)}{\hat{l}\left(z\right)}\right]$ where *ln* stands for natural logarithm, Î(*z*) is the likelihood function evaluated at the maximum likelihood estimate (MLE) for the full model that includes polygenic and QTL effects, and Î_*r*_(*z*) is the MLE for the restricted model under which *r* parameters of the full model are assigned fixed values [36]. The *P*-value was calculated assuming an *F*-value distributed with numerator DF equal to the number of sires or dams, and denominator DF equal to the total number of offspring minus twice the number of sires or dams [35]. The chromosome-wide *F*-value (*F*_*ChromWide P=0.05*_) and experiment-wide *F*-value (*F*_*ExperWide P*=0.05_) were estimated using 10,000 permutations with software GridQTL [34]. The genome-wide significance level (*P*_*GenomeWide*_) for detected QTL was estimated as *P*_*GenomeWide*_ = 1 − (1 − *P*)^*g*^[37] where *P* is the nominal *P*-value, and *g* = 380 STR loci used in the HS regression analysis. The QTL with *F*-value ≥ *F*_*ChromWide P=0.05*_ was defined as suggestive QTL (*); and QTL with *F*-value ≥ *F*_*ExperWide P=0.05*_ or *P*_*GenomeWide*_ ≤ 0.05 was defined as significant QTL (**). The proportion of phenotypic variance explained by the QTL was calculated as $h\frac{2}{q}=4\;\left[1-\left(MSE_{\mathrm{full}}/MSE_{\mathrm{reduced}}\right)\right]$ where *MSE*_*full*_ and *MSE*_*reduced*_ are the mean squared error of the full and reduced model, respectively [35]. The 95% QTL confidence intervals were estimated using 10,000 bootstraps with re-sampling with software GridQTL [34].

Briefly, the HS regression analysis was performed following these steps: First, all chromosomes were genome scanned for EBV/BLUP3 QTL performing sire-family and dam-family regression analysis, separately, using one-QTL model. At this stage: (a) the chromosome-wide significance threshold level (*F*_*chromWideP*=0.05_) to declare suggestive QTL was determined using 10,000 permutations; and (b) the 95% confidence interval (CI95) for each suggestive QTL was determined using 10,000 bootstraps with re-sampling. Second, chromosomes with suggestive QTL were re-scanned for additional QTL using one-QTL model that accounted for the effect of already detected QTL until not detecting more QTL in a chromosome. Third, the chromosomes with two detected QTL were re-scanned using two-QTL models. In the chromosomes with two detected QTL: (a) the (*F*_*chromWideP*=0.05_) significance level for one QTL at a time was estimated while accounting for the effect of the other detected QTL using 10,000 permutations; and (b) the CI95 for each QTL was determined by fixing alternatively the effect of each detected QTL using 10,000 bootstraps with re-sampling [38]. Fourth, the experiment-wide significance threshold level (*F*_*ExperWideP*=0.05_) to declare significant QTL was determined by analyzing altogether marker genotype data from 29 chromosomes using one-QTL models and 10,000 permutations [25,26].

### QTL analysis using variance components approach

We also performed a genome scan for genetic loci linked to stress response phenotypes (animal BLUP3 index and EBV for plasma cortisol) using a variance components (VARCOMP) method of QTL analysis with software SOLAR version 4.0 [39]. The aim on using a second linkage-based method of QTL analysis was to reduce the rate of false positive claims of QTL. This robust VARCOMP method of QTL analysis was performed using the seven FS families altogether (total parents and offspring *n* =234). This combined family QTL analysis enables accounting for the impact of common environmental effects on QTL mapping which increases the resemblance between full-sibs as they share the same environment during early stages of rainbow trout rearing.

In order to perform multipoint QTL analysis, the multipoint identical by descent (MIBD) relationship matrix for each pair of individuals was estimated with the program LOKI version 2.4.5 [40]. We used LOKI because SOLAR cannot estimate MIBDs in complex pedigrees with double grandparent-grandchild relationships as those used in this study. The VARCOMP approach performs multipoint genome scan at 1-cM intervals. At each testing interval, SOLAR calculates the residual genetic variance or proportion of the total variance due to the polygenic component (*h*_*u*_^2^); the heritability associated with the QTL or proportion of the total variance due to the QTL (*h*_*q*_^2^); and the logarithm of odds (LOD) score as *LOD* = *log*_10_*L*(*QTL*)/*L*(*polygenic*)], where *L(QTL)* stands for the likelihood of the full model that includes the polygenic and QTL effect. The genome-wide significance level of detected QTL was estimated using this expression *P*_*GenomeWide*_ = 1 − (1 − *P*)^*g*^[37] which assumes the use of sparse genetic maps; here *P* is the nominal *P*-value, and g = 365 STR loci used with VARCOMP analysis (average marker distance = 6.6 cM). The QTL with LOD ≥ 2 was defined as suggestive QTL (*); and the QTL with LOD ≥ 3 or *P*_*GenomeWide*_ ≤ 0.05 was defined as significant QTL (**).

### QTL analysis using LDLA method

We performed a genome scan for stress response QTL using an LDLA method with the module from the web-based software GridQTL [34,41]. We reasoned that the rate of false positive QTL may be reduced by following up the linkage-based QTL analyses with an LDLA-based method of QTL analysis because the QTL signals have to conform with both LD and LA assumptions [42].

The LR test statistic is defined as indicated above in the section of QTL mapping using HS regression analysis. The LDLA method performs LR tests at 1-cM intervals along the chromosome. Here, the LR test statistic is defined as *LR* = *L*(*QTL*)/*L*(*polygenic*) where *L(QTL)* is the likelihood of the full model that includes the polygenic and QTL effects. The nominal *P*-value was estimated assuming the LR test statistic follows a chi-square distribution with two degrees of freedom [43]. The genome-wide significance level of detected QTL was also estimated as *P*_*GenomeWide*_ = 1 − (1 − *P*)^*g*^[37] where *P* is the nominal *P*-value, and g = 365 STR loci used with LDLA analysis. The QTL with LR ≥ 13.82 or nominal *P* ≤ 0.001 was declared as suggestive QTL (*), and the QTL with *P*_*GenomeWide*_ ≤ 0.05 was declared as significant QTL (**). The variance components due to polygenic (*σ*_*u*_^2^), additive QTL (*σ*_*a*_^2^), dominant QTL (*σ*_*d*_^2^) and residual error (*σ*_*e*_^2^) were used to estimate the proportion of total variance due to the polygenic component *h*_*u*_^2^ = *σ*_*u*_^2^/(*σ*_*u*_^2^ + *σ*_*a*_^2^ + *σ*_*d*_^2^ + *σ*_*e*_^2^)], the proportion of total variance due to additive effect QTL *h*_*addQTL*_^2^ = *σ*_*a*_^2^/(*σ*_*u*_^2^ + *σ*_*a*_^2^ + *σ*_*d*_^2^ + *σ*_*e*_^2^)], and the proportion of total variance due to dominance effect QTL *h*_*domQTL*_^2^ = *σ*_*d*_^2^/(*σ*_*u*_^2^ + *σ*_*a*_^2^ + *σ*_*d*_^2^ + *σ*_*e*_^2^)], respectively.

## Results

### Correlations among repeated measurements

The heritability, genetic and phenotypic correlations between the four plasma cortisol measurements are presented in Table 1. Heritability estimates for the first measurements were significantly less than the second through fourth measurements, with the heritability increasing over time 1 to 4. The ranges of genetic and phenotypic correlations were much higher when the first measurement is not included.

**Table 1:** **Quantitative genetic analysis of plasma cortisol repeated measurements**^**1**^**in F**_**1**_**QTL mapping families of rainbow trout**

**Repeated measurement**	**Cortisol 1**	**Cortisol 2**	**Cortisol 3**	**Cortisol 4**
**Cortisol 1**	**0.0819 ± 0.0903**^2^	0.4311 ± 0.0588^4^	0.1999 ± 0.0735	0.1802 ± 0.0737
**Cortisol 2**	0.5484 ± 0.3868^3^	**0.3083 ± 0.1209**	0.4442 ± 0.0633	0.4779 ± 0.0609
**Cortisol 3**	0.1852 ± 0.5172	0.8456 ± 0.1439	**0.4599 ± 0.1489**	0.4484 ± 0.0671
**Cortisol 4**	0.2002 ± 0.4871	0.9184 ± 0.0980	0.9580 ± 0.0869	**0.5015 ± 0.1345**

^1^The measurement unit is expressed in ng/mL of plasma cortisol.

^2^The heritability estimates with their corresponding S.E. are bold text highlighted and shown in the main diagonal.

^3^The genetic correlation estimates with their corresponding S.E. are shown in the lower diagonal.

^4^The phenotypic correlation estimates with their corresponding S.E. are shown in the upper diagonal.

### Loci testing for Mendelian segregation distortion

Testing 346 short tandem repeat (STR) loci for Mendelian segregation distortion (MSD) resulted in about 1%, 6%, 4%, 2%, 2%, 3% and 2% of markers having significant MSD (*P* ≤ 0.01) in families 1, 2, 3, 4, 5, 6 and 7, respectively (Additional file 1). From all markers with significant MSD, four of these markers were flanking suggestive QTL for stress response: OMM1130 (Omy12); OMM5153 (Omy14); BX913059 (Omy22); and OMM1772 (OmySex) (Tables 2 and 3).

**Table 2:** Results of a genome scan for QTL associated with stress response using half-sib regression interval mapping

**Omy**	**cM**	**Trait**	**Sire-dam family**^**4**^	**LR**^**1**^	***F*-value**^**2**^	***F***_***ChromWide P=0.05***_^**3**^	***F***_***ExperWide P=0.05***_^**4**^	***P-value***^**5**^	***P***_***GenomeWide ***_^**6**^	***h***_***q***_^***2***^^**7**^	**Left flanking marker**	**Right flanking marker**
6	32.0	BLUP3	Dam	21.14	3.14*	2.85	4.16	0.00350	0.736	0.26	OMY105DU	OMM5254
10	70.0	BLUP3	Dam	18.41	2.72	2.91	4.16	0.01002	0.978	0.21	OMM1544	OMM5108
10	72.0	EBV	Dam	23.19	3.46*	2.88	4.15	0.00155	0.445	0.30	OMM1544	OMM3102
12	36.0	EBV	Dam	19.83	2.93*	2.75	4.15	0.00594	0.896	0.24	OMM1096	OMM1130^8^
12	60.0	EBV	Sire	19.88	4.14*	2.71	4.48	0.00129	0.388	0.27	OMM1341	OMM1711
14	95.0	EBV	Sire	15.8	3.26*	2.78	4.48	0.00733	0.939	0.20	OMM1643	OMM5153^8^
16	45.0^9^	BLUP3	Sire	17.4	3.60*	2.59	4.53	0.00108	0.336	0.23	OMM1559	OMM5162
16	65.0	BLUP3	Dam	20.44	3.03*	2.77	4.16	0.00462	0.828	0.25	OMM1150	OMM1221
16	71.0^9^	BLUP3	Sire	22.64	4.75**	2.61	4.53	0.00005	0.020	0.32	OMM1150	OMM1221
19	40.0	EBV	Sire	14.33	2.94*	2.93	4.48	0.01365	0.995	0.17	OMM1241	OMM3067
19	45.0	BLUP3	Sire	14.59	3.00*	2.95	4.53	0.01215	0.990	0.18	OMM1241	OMM3067
19	64.0	BLUP3	Dam	18.34	2.70	2.77	4.16	0.01052	0.982	0.21	OMM5327	OMM5216
22	35.0	EBV	Dam	19.61	2.90*	2.54	4.15	0.00640	0.913	0.23	OMM1292	BX913059^8^
29	50.0^10^	EBV	Sire	14.76	3.04*	2.73	4.48	0.01125	0.986	0.18	OMM1772^8^	OMM1118
29	56.0^10^	EBV	Sire	14.6	3.01*	2.70	4.48	0.01192	0.990	0.18	OMM1118	OMM1405

^1^The likelihood ratio (LR) test statistic.

^2^Asymptotically *F-*test statistic with the degrees of freedom(DF) being the number of sires or dams included for the numerator, and the total number of offspring minus twice the number of sires or dams for the denominator. The QTL with *F*-value ≥ *F*_*ChromWide P=0.05*_ was defined as suggestive QTL (*); and QTL with *F*-value ≥ *F*_*ExperWide P=0.05*_ or *P*_*GenomeWide*_ ≤ 0.05 was defined as significant QTL (**).

^3^Chromosome-wide *F*-value at *P*=0.05 was estimated using 10,000 permutations with software GridQTL.

^4^Experiment-wide *F*-value at *P*=0.05 was estimated using 10,000 bootstraps with re-sampling with software GridQTL.

^5^The nominal *P*-value was calculated assuming an *F*-value distributed with numerator DF equal to the number of sires or dams, and denominator DF equal to the total number of offspring minus twice the number of sires or dams.

^6^The genome-wide significance level for detected QTL.

^7^The proportion of phenotypic variance explained by the QTL.

^8^The QTL flanking markers OMM1772, OMM1130, BX913059 and OMM5153 had significant Mendelian segregation distortion (*P* <0.01) in families 2, 4, 5 and 6, respectively.

^9^The Omy16 QTL had significant statistical support for two QTL segregating (*F*_2*QTL vs.* 0*QTL; DF*=10_=3.58; *F*_1*QTL vs.* 0*QTL; DF*=5_=3.60; *LR*_2*QTL vs.* 0*QTL*_ = 33.62) for BLUP3 when fitting a two-QTL model with sire HS families.

^10^The Sex chromosome QTL had significant statistical support for two QTL segregating (*F*_2*QTL vs.* 0*QTL; DF*=10_=3.45; *F*_1*QTL vs.* 0*QTL; DF*=5_=3.10; *LR*_2*QTL vs.* 0*QTL*_ = 32.50) for EBV when fitting a two-QTL model with sire HS families.

**Table 3:** Results of a genome scan for QTL associated with stress response using a variance components approach

**Omy**	**cM**	**Trait**	**LOD score**^**1**^	***P***	***P***_***GenomeWide***_^**2**^	***h***_***u***_^**2**^^**3**^	***h***_***q***_^**2**^^**4**^	**Left flanking marker**	**Right flanking marker**
3	116	BLUP3	0.9	0.02394	1.000	0.00	0.16	OMM1263	OMM1391b
4	112	BLUP3	0.9	0.02012	0.999	0.00	0.19	OMM1582	OMM1408^5^
4	112	EBV	0.9	0.02012	0.999	0.00	0.17	OMM1582	OMM1408^5^
9	77	BLUP3	1.0	0.01470	0.996	0.00	0.23	OMM1089	OMM5054
10	52	EBV	0.7	0.03736	1.000	0.01	0.46	OMM1549b	OMM5312
12	39	EBV	**1.9**	**0.00150**	0.421	0.01	0.24	OMM1096	OMM1130^5^
14	119	EBV	0.9	0.02155	1.000	0.01	0.36	OMM5153	OMM5143^5^
16	69	BLUP3	3.3**	**0.00005**	**0.018**	0.00	0.52	OMM1150	OMM1221
17	46	EBV	1.1	0.01072	0.980	0.01	0.49	OMM5026	OMM3027
19	75	BLUP3	1.2	0.01069	0.980	0.00	0.14	OMM1739	OMM5106a^5^
19	114	EBV	1.0	0.01464	0.995	0.01	0.38	OMM1549a	OMM1124b
22	37	EBV	0.7	0.04031	1.000	0.01	0.12	OMM1457	BX913059^5^
23	31	EBV	1.1	0.01373	0.994	0.01	0.39	OMM5305	OMM1623
28	58	EBV	0.9	0.02013	0.999	0.01	0.36	OMY1013UW	OMYRGT51TUF
Sex	38	EBV	**1.6**	**0.00371**	0.743	0.01	0.43	OMM1715a	BX076085

^1^Logarithm of odds (LOD) score was calculated as *LOD* = *log*_10_[*L*(*QTL*)/*L*(*polygenic*)] where L = likelihood of the model. QTL with LOD ≥ 2 was defined as suggestive QTL (*); and QTL with LOD ≥ 3 or *P*_*GenomeWide*_ ≤ 0.05 was defined as significant QTL (**).

^2^The genome-wide significance level for detected QTL was estimated as *P*_*GenomeWide*_ = 1 − (1 − *P*)^*g*^, where *P* is the nominal *P*-value, and g = 365 STR loci used with variance components method of QTL analysis.

^3^*h*_*u*_^2^ is the residual genetic variance or proportion of the total variance due to the polygenic component.

^4^*h*_*q*_^2^ is the heritability associated with the QTL or proportion of the total variance due to the QTL.

^5^The QTL flanking markers OMM5106a, OMM1408, OMM1130, BX913059 and OMM5143 had significant Mendelian segregation distortion (*P* <0.01) in families 2, 3, 4, 5 and 6, respectively.

### Genotyping and linkage analysis

The genetic linkage analyses resulted in the construction of genetic maps representing 29 chromosomes and including 380 microsatellite markers whose orders were found to be in good agreement with markers on the NCCCWA genetic map [19]. Marker densities ranged from 4.5 to 10.9 cM per chromosome with a genome average of 6.4 cM spanning 2426 cM. A summary of genetic maps for each chromosome including marker information content across sires and dams is presented in Table 4.

**Table 4:** Genome scan linkage mapping information per chromosome

**Chromosome (Omy)**	**Number of markers**	**Length (cM)**	**Average marker density**	**Marker information content**^**1**^				**HS**^**2**^		**VC**^**3**^		**LDLA**^**4**^	
				**Dam families**		**Sire families**							
				**Mean**	**SD**	**Mean**	**SD**	**EBV**	**BLUP3**	**EBV**	**BLUP3**	**EBV**	**BLUP3**
1	14	63.2	4.5	0.79	0.06	0.88	0.10						
2	13	100.2	7.7	0.71	0.10	0.80	0.17						
3	12	112.3	9.4	0.74	0.13	0.80	0.09					**	*
4	13	111.1	8.5	0.71	0.15	0.75	0.16					**	
5	13	101.5	7.8	0.77	0.11	0.80	0.15					**	**
6	17	92.0	5.4	0.82	0.07	0.87	0.13		*			**	
7	19	133.1	7.0	0.78	0.17	0.78	0.18					**	
8	18	103.0	5.7	0.84	0.10	0.80	0.09					**	
9	17	104.0	6.1	0.74	0.11	0.88	0.09					**	
10	20	117.5	5.9	0.79	0.15	0.88	0.12	*				**	
11	13	85.2	6.6	0.74	0.07	0.75	0.10					**	
12	16	78.7	4.9	0.76	0.09	0.91	0.06	*		*		**	
13	9	65.8	7.3	0.69	0.05	0.94	0.04					**	*
14	18	103.9	5.8	0.74	0.17	0.82	0.17	*				*	
15	12	61.2	5.1	0.80	0.08	0.81	0.10						
16	13	72.0	5.5	0.81	0.08	0.95	0.03		**		**	**	**
17	19	110.3	5.8	0.86	0.06	0.91	0.09					**	
18	10	108.5	10.9	0.65	0.13	0.60	0.11					*	
19	13	115.7	8.9	0.68	0.08	0.74	0.09	*	*			**	
20	8	42.8	5.4	0.81	0.07	0.87	0.04						
21	14	72.4	5.2	0.82	0.08	0.92	0.09					*	
22	8	62.7	7.8	0.76	0.07	0.74	0.08	*					
23	6	28.7	4.8	0.86	0.07	0.88	0.06						
24	8	37.4	4.7	0.82	0.08	0.84	0.03						
25	19	105.1	5.5	0.80	0.09	0.84	0.11					**	
26	9	65.4	7.3	0.70	0.10	0.74	0.17					**	
27	11	58.1	5.3	0.88	0.05	0.77	0.17					**	
28	9	53.0	5.9	0.83	0.09	0.79	0.09					**	
Sex	9	60.9	6.8	0.75	0.05	0.88	0.12	*		*		**	*
**Total/average**	**380**	**2425.7**	**6.4**	**0.77**		**0.83**		**6/0**^**5**^	**2/1**	**2/0**	**0/1**	**3/19**	**3/2**

^1^Marker information content combines the multilocus probability of individuals inheriting allele 1 or 2 from the common parent with marker segregation distortion [35].

^2-4^The HS, VC, and LDLA columns show chromosomes containing suggestive (*) and significant (**) QTL for each trait (EBV, BLUP3) identified by half-sib regression interval mapping, variance component analysis, or linkage disequilibrium and linkage analysis, respectively.

^5^The number of total QTL is reported in the last row in the format suggestive/significant QTL.

### Identification of QTL using half-sib regression interval mapping

Results of QTL analyses for EBV and BLUP3 using half-sib regression interval mapping (HS) are presented in Table 2. Six suggestive QTL were detected for EBV (Omy10, 12 14, 19, 22 and Sex) which each explain 17 - 30% of the phenotypic variance. For BLUP3, suggestive (Omy6 and 19) and one significant (Omy16) QTL were detected that explain 18-32% of the phenotypic variance. The only overlapping QTL between traits are suggestive QTLs on Omy19. Multiple QTL were detected on Omy12, 16, 19 and Sex.

On chromosomes with two detected QTL using one-QTL models, we found significant statistical support for two QTL segregating on Omy16 (BLUP3BLUP33; *F*_2*QTL vs.* 0*QTL; DF*=10_=3.58; *F*_1*QTL vs.* 0*QTL; DF*=5_=3.60; *LR* = 33.62) and Sex chromosome (EBV; *F*_2*QTL vs.* 0*QTL; DF*=10_=3.45; *F*_1*QTL vs.* 0*QTL; DF*=5_=3.58; *LR* = 32.50) when fitting two-QTL models with sire HS families (Table 2). The estimated sire QTL effects with their corresponding significance level suggest that Sire 5 and 3 were likely segregating the QTL detected on Omy16 and Sex, respectively (Additional file 2).

### Identification of QTL using a variance components approach

Results of the QTL analyses using the variance component (VC) approach are presented in Table 3. Two suggestive QTL explaining 24 and 43% of the phenotypic variance were detected for EBV (Omy12 and Sex) and a significant QTL explaining 52% of the phenotypic variation was detected for BLUP3BLUP33 (Omy16). Both of the QTL for EBV overlap with similar QTL detected with HS regression interval mapping (Table 2). The significant QTL for BLUP3 on Omy16 was also determined to be significant by HS regression analyses (Table 2).

### Identification of QTL using linkage disequilibrium and linkage analysis

Results of efforts to detect QTL for EBV and BLUP3 using linkage disequilibrium and linkage analysis (LDLA) are presented in Table 5. Three suggestive (Omy14, 18, and 21) and 19 significant (Omy3-13, 16, 17, 19, and 25-Sex) QTL were detected for EBV; three suggestive (Omy3, 13, and Sex) and two significant (Omy5 and 16) QTL were detected for BLUP3.

**Table 5:** Results of a genome scan for QTL associated with stress response using linkage disequilibrium and linkage analysis

**Omy**	**cM**	**Trait**	**LR**^**1**^	***P***^**2**^	***P***_***GenomeWide ***_^**3**^	**LOD score**^**4**^	***h***_***u***_^**2**^^**5**^	***h***_***addQTL***_^**2**^^**6**^	***h***_***domQTL***_^**2**^^**7**^	**Flanking markers**	
										**Left**	**Right**
3	79.0	EBV	173.77**	1.8E-38	6.6E-36	36.2	0.00	0.00	0.98	OMM3120	OMM5109
3	98.0	BLUP3	16.65*	2.4E-04	8.5E-02	2.6	0.00	0.00	0.84	OMM1263	OMM1391b
4	7.0	EBV	39.75**	2.3E-09	1.0E-06	7.5	0.00	0.03	0.81	OMM1194^8^	OMM1211
5	90.0	BLUP3	58.23**	2.3E-13	8.3E-11	11.4	0.00	0.76	0.11	OMM5025	OMM1774
5	99.0	EBV	20.63**	3.3E-05	1.2E-02	3.5	0.00	0.00	0.70	OMM5025	OMM1774
6	67.0	EBV	53.03**	3.1E-12	1.1E-09	10.3	0.00	0.00	0.71	OMM1753	OMM1628
7	35.0	EBV	31.27**	1.6E-07	5.9E-05	5.7	0.43	0.00	0.48	OMM1468	OMM1740^8^
8	55.0	EBV	46.24**	9.1E-11	3.3E-08	8.8	0.00	0.37	0.29	OMM1295	OMM1632
9	71.0	EBV	18.83**	8.1E-05	2.9E-02	3.1	0.00	0.10	0.36	OMM5179	OMM1089
10	100.0	EBV	81.7**	1.8E-18	6.6E-16	16.4	0.00	0.00	0.64	CR372971^8^	OMY1000UW
11	34.0	EBV	22.36**	1.4E-05	5.1E-03	3.8	0.00	0.00	0.61	OMM1333	OMM3042
12	33.0	EBV	50.87**	9.0E-12	3.3E-09	9.8	0.52	0.00	0.38	OMY105INRA	OMM1096
13	50.0	EBV	26.45**	2.0E-06	6.6E-04	4.7	0.00	0.00	0.54	OMY1UoG	OMM5165a
13	51.0	BLUP3	15.05*	5.4E-04	1.8E-01	2.3	0.16	0.00	0.35	OMY1UoG	OMM5165a
14	46.0	EBV	16.05*	3.3E-04	1.1E-01	2.5	0.14	0.00	0.47	OMM1038	OMM1415
16	54.0	EBV	21.32**	2.3E-05	8.5E-03	3.6	0.18	0.13	0.34	OmyRGT6TUFa	OMM1362
16	69.0	BLUP3	22.89**	1.1E-05	3.9E-03	3.9	0.00	0.61	0.00	OMM1150	OMM1221
17	75.0	EBV	49.57**	1.7E-11	6.3E-09	9.5	0.00	0.45	0.23	OMM3126	OMM1808
18	108.0	EBV	16.64*	2.4E-04	8.5E-02	2.6	0.48	0.00	0.42	OMM1352	BX873238
19	84.0	EBV	45.14**	1.6E-10	5.8E-08	8.6	0.47	0.00	0.46	OMM5106a^8^	OMM1412b
21	16.0	EBV	17.23*	1.8E-04	6.4E-02	2.8	0.00	0.00	0.54	OMM1256^8^	OMM5298
25	0.0	EBV	19.97**	4.6E-05	1.7E-02	3.3	0.00	0.00	0.68	OMM3142	BX881655a
26	56.0	EBV	29.97**	3.1E-07	1.1E-04	5.4	0.00	0.00	0.83	OMY1189UWa	OMM1752b
27	28.0	EBV	19.3**	6.4E-05	2.3E-02	3.2	0.33	0.00	0.49	OMM1315^8^	OMM5309
28	48.0	EBV	19.71**	5.2E-05	1.9E-02	3.3	0.00	0.00	0.53	CR373404^8^	OMY1013UW
Sex	20.0	EBV	53.66**	2.2E-12	8.1E-10	10.4	0.00	0.28	0.48	OMM1026	OMM1461
Sex	44.0	BLUP3	16.56*	2.5E-04	8.8E-02	2.6	0.47	0.00	0.38	BX076085	OMM1772^8^

^1^The likelihood ratio (LR) test statistic is defined as $LR=-2ln\left[\frac{{\hat{l}}_{r}\left(z\right)}{\hat{l}\left(z\right)}\right]$ where *ln* stands for natural logarithm, Î(*z*) is the likelihood function evaluated at the maximum likelihood estimate (MLE) for the full model that includes polygenic and QTL effects, and Î_*r*_(*z*) is the MLE for the restricted model under which *r* parameters of the full model are assigned fixed values. The QTL was declared as suggestive if it had a LR ≥ 13.82 or nominal *P* ≤ 0.001 (*), and as significant if it had a *P*_*GenomeWide*_ ≤0.05 (**).

^2^The nominal *P*-value was estimated assuming the LR test statistic follows a chi-square distribution with two degrees of freedom.

^3^The genome-wide significance level for detected QTL was estimated as *P*_*GenomeWide*_ = 1 − (1 − *P*)^*g*^[37] where *P* is the nominal *P*-value, and g = 365 STR loci used with LDLA method of QTL analysis.

^4^Logarithm of odds (LOD) score was calculated as *LOD* = *log*_10_[*L*(*QTL*)/*L*(*polygenic*)] where L = likelihood of the model.

^5^*h*_*u*_^2^ is the residual genetic variance or proportion of the total variance due to the polygenic component.

^6^*h*_*addQTL*_^2^ is the proportion of the total variance due to additive effect QTL.

^7^*h*_*domQTL*_^2^ is the proportion of the total variance due to dominance effect QTL.

^8^These QTL flanking markers had significant Mendelian segregation distortion (*P* <0.01): OMM1194, OMM1740, OMM5106a and OMM1772 in family 2; CR372971 and CR373404 in family 3; OMM1256 in family 4; OMM1315 in family 5; and OMM1752b in family 7.

## Discussion

Identifying genetic variation in mechanisms underlying complex traits such as response to stress may improve our ability to mitigate the negative effects of stressors on aquaculture production through selective breeding or management practices. To this end we sought to identify QTL affecting response to crowding in seven families from a broodstock population under selection for increased growth rate for a single generation. The families evaluated in this study were not bred specifically for QTL analyses; therefore multiple approaches to QTL discovery were performed as a means of validation and to identify an inclusive list of chromosomes with genes affecting stress phenotypes. Although the 7 families including 5 sires, 7 dams and a total of 222 offspring is low for estimating heritability and genetic correlations, we were able to detect a single significant and 7 suggestive QTL. Although we have detected locations of QTL at the sub-chromosomal level on the genetic maps, it is possible that multiple QTL on a single chromosome are actually the same QTL identified in different families and map positions differ because of imbalances in female:male recombination ratios [44]. Therefore, we take a conservative approach and focus our discussion at the level of the chromosome. These results are summarized in Table 4, where suggestive and significant QTL are tabulated according to each analysis and trait.

### Comparisons of EBV and BLUP3

In estimating the heritability and genetic correlation between repeated plasma cortisol measurements, we observed that measurement at time point 1 was markedly different in terms of heritability and genetic correlations than those measurements at times 2, 3 and 4 which were all about a month apart. This may indicate that physiological response to stress is altered after the initial challenge and genes participating in a first response to a stressor might differ in part from that of subsequent exposures. Overall our interest is in the responses of rainbow trout to the stressors of the aquaculture production environment throughout the entire grow-out cycle, therefore we sought to identify a robust trait which characterizes the chronic response as closely as possible. Given our observation that the measurements taken at the first time point was somewhat of an outlier; we developed BLUP3 using only measurements at time points 2, 3 and 4 weighted by their corresponding relative heritability. Our thought was there might be something unique and biologically interesting about the first measurement such that not including it would result in the detection of different sets of QTL than when all four measurements are included (EBV). In fact, the HS analysis resulted in the detection of QTL for both traits on Omy19, with five chromosomes having QTL specific for EBV and two specific for BLUP3. Three QTL detected by VC analysis were trait specific. Eventually we hope to realize the impacts these QTL have on production traits and determine if one or the other is a more accurate predictor of long term response to stress.

### Comparisons of QTL detected by HS, LDLA, and VC

The three methods of QTL analysis (HS, LDLA and VC) used in this study detected unique sets of QTL along the scanned rainbow trout genome due to several reasons. First, each of these methods uses a different algorithm of QTL analysis. The HS and VC methods are linkage-based methods, and the LDLA method exploits simultaneously linkage and LD information from the sampled families. The HS method uses a least-square based regression interval mapping approach, and the VC method is a variance components based approach. From others and our extensive testing of these computer packages, we noticed that the VC algorithm is more conservative and robust to violations of assumptions on trait multivariate normal distribution with low rate of type I errors in the analysis of complex traits [39,45]. In contrast, the HS and LDLA methods seem to be more liberal and powerful than the VC method with a trade-off of more type I errors in the HS and LDLA engines. Clearly, the test-statistic values were generally larger for the HS and LDLA methods than for those of the VC method. Comparing the test-statistics of these three methods of QTL analysis, we noticed that they display a similar trend along the genome although at different scale of magnitude: higher scale of magnitude for the HS and LDLA method, and lower scale of magnitude for the VC method; consequently reaching most often statistical significance with the HS and LDLA method, and less often with the VC method. Coupling with complementing use of EBV and BLUP3 to estimate the effects of initial vs. repeated stressors, we believe that this strategy of using three different algorithms of QTL mapping allowed us to reduce the false positive rate of detected QTL in this genome scan. To this end across the three analyses significant QTL were detected on Omy16 for BLUP3; and at least suggestive QTL for EBV were detected on Omy12 and Sex. However, we acknowledge that there is also a likelihood of false negatives as these QTL detection methods are not optimized for our mapping families, and that lack of validation across analyses does not necessarily invalidate QTL detected in a single analyses.

LDLA detected QTL for EBV on 22 chromosomes and for BLUP3 on 5 chromosomes, identifying a high degree of false positives. The robustness of LDLA software has not yet been extensively tested, and the LDLA developers warned about the potential pitfalls [41]. They indicated that a large significant result obtained with LDLA and a very insignificant result obtained with LA method may indicate a false positive result. So, here the LDLA results were handled with caution and compared with HS and VC results.

Drew *et al.*[11] compared response to netting stress in three clonal lines of rainbow trout differing in their histories of domestication and observed significant variation in plasma cortisol concentrations. Genotyping of doubled haploid offspring created from crossing two of those strains identified two QTL with significant and antagonistic additive effects that explained 43% of the phenotypic variance, one of which overlapped with a QTL for juvenile body mass. Our results from HS and VC do not overlap with the findings of that study. However, given that the stressor was not exactly the same in the two experiments and the differences in populations, family structures, and analytical methods; it is possible there exists some overlap in QTL affecting stress response in the populations from both studies that were just not detected due to differences in experimental designs. The number of QTL identified across studies highlights the large degree of genetic variation affecting stress responses in rainbow trout.

### Comparing results with CSA

Performing CSA for stress response traits, we found that more than one major gene with dominant cortisol-decreasing alleles and also major genes with additive effect of −42 ng/mL were segregating in the NCCCWA rainbow trout broodstock population [17]. The results found in this QTL genome scan partially validate the previous CSA report. Here, using linkage-based methods of QTL analysis (HS and VC), we identified eight suggestive/significant QTL that were validated with LDLA method. These detected QTL explained a wide range of phenotypic trait variation (17-52%). The additive effect of suggestive/significant QTL detected with HS regression analysis ranged from -17.98 to 17.0 ng/mL (Additional file 2). However, the additive effect of significant QTL detected on Omy16 was -17.9 ng/mL which even as a cumulative effect was remarkably smaller than the predicted effect of the CSA QTL.

By design, the HS regression analysis performed with GridQTL [34] can make inferences only about additive genetic effects. Although we performed combined FS family analysis with the VC method, the SOLAR software was implemented to fit only additive effect QTL models. In contrast, the combined FS family analysis performed with the LDLA method allowed testing for additive and dominance QTL effects. From the QTL detected with the LDLA method (Table 5) about 1/3 and 2/3 of detected QTL had additive and dominance effects, respectively.

## Conclusions

We report the detection of multiple QTL for stress response as defined by Pottinger and Carrick [18] and modified by Weber and Silverstein [12] in a mapping population sampled from broodstock under selection for growth. Suggestive and significant QTL affecting stress responses were detected on eight chromosomes in a survey of seven families from a single broodstock population. These QTL are largely different from others previously identified for plasma cortisol level in response to stress in rainbow trout, implying that many genetic variants may affect this trait which may independently affect other traits. Also, mapping QTL for EBV and BLUP3 produced trait specific QTL which indicate the significance of the first measurement in defining traits associated with stress response. Validation of these QTL in other families and populations followed by fine mapping will lead towards the identification of genes affecting stress response and may influence approaches to selection regarding these economically important traits.

## Authors' contributions

CER and GMW designed overall study including the breeding scheme, GMW executed the stress challenge and phenotyping, RLV performed QTL analyses, and SL and YP participated in genotyping and linkage analysis. All authors read and approved the final manuscript.

## Supplementary Material

Additional file 1 **STR loci testing for Mendelian segregation distortion (MSD).** In outbred populations, the progeny of an informative QTL mapping family can have any of these marker genotype proportions: 1:1; 1:2:1 and 1:1:1:1. Within each of seven FS families, the marker genotype counts were performed using a Perl script (Written by G. GAO, unpublished). Then , the STR loci were tested for MSD using Chi-Square goodness-of-fit test of marker genotype counts to expected proportions under Mendelian segregation with SAS Procedure FREQ (SAS, 2007) using a default significance level of α = 0.01. Click here for file

Additional file 2 **QTL location and effect for stress response trait0073.** Summary of QTL location and effect for stress response traits using combined sire (and dam) half-sib family regression analysis performed with software GridQTL (Seaton *et al.* 2006). The QTL effect is expressed in ng/mL of plasma cortisol, and the allele substitution effect for each parent was tested using a one-sided t-test (testing absolute *t*-values) with one DF. Within each trait QTL group, the sire or dam parent that is most likely segregating the QTL allele is indicated with an asterisk (*), and the parent with the lowest *P*-value is bold text highlighted. The average QTL location was determined using 10000 bootstraps with re-sampling. Click here for file
