# Supplementary material for: QTL affecting stress response to crowding in a rainbow trout broodstock population
Source: BMC Genet. 2012 Nov 7;13:97. doi: 10.1186/1471-2156-13-97 (PMC3531310; doi:10.1186/1471-2156-13-97)
Supplement: Additional file 2 — QTL location and effect for stress response trait0073. Summary of QTL location and effect for stress response traits using combined sire (and dam) half-sib family regression analysis performed with software GridQTL (Seaton et al. 2006). The QTL effect is expressed in ng/mL of plasma cortisol, and the allele substitution effect for each parent was tested using a one-sided t-test (testing absolute t-values) with one DF. Within each trait QTL group, the sire or dam parent that is most likely segregating the QTL allele is indicated with an asterisk (*), and the parent with the lowest P-value is bold text highlighted. The average QTL location was determined using 10000 bootstraps with re-sampling. [file 1471-2156-13-97-S2.docx]

**Additional File 2** Location and effect of suggestive/significant QTL for stress response traits performing combined sire (or dam) half-sib family regression analysis with software GridQTL^1^

| **OMY^2^** | **Trait^3^** | **cM^4^** | **Parent^5^** | **Animal ID** | **QTL effect^6^** | **SE** | **Abs *t*-value** | ***P*-value^7^** |
| --- | --- | --- | --- | --- | --- | --- | --- | --- |
| 6 | Blup | 32.0 | Dam 1 | 133565797 | -4.95 | 4.33 | 1.14 | 0.23 |
| 6 | Blup | 32.0 | Dam 2 | 134919497 | -0.74 | 4.15 | 0.18 | 0.44 |
| 6 | Blup | 32.0 | Dam 3 | 134534543 | 5.23 | 3.67 | 1.43 | 0.19 |
| 6 | Blup | 32.0 | Dam 4 | 134633225 | 3.31 | 3.50 | 0.94 | 0.26 |
| 6 | Blup | 32.0 | Dam 5 | 134567133 | 2.49 | 3.74 | 0.66 | 0.31 |
| 6 | Blup | 32.0 | Dam 6 | 134626172 | 3.04 | 3.30 | 0.92 | 0.26 |
| 6 | Blup | 32.0 | Dam 7* | 134561470 | **15.83** | 3.91 | 4.05 | **0.08** |
| 10 | Blup | 70.0 | Dam 1 | 133565797 | -2.65 | 5.50 | 0.48 | 0.36 |
| 10 | Blup | 70.0 | Dam 2 | 134919497 | -2.37 | 3.52 | 0.67 | 0.31 |
| 10 | Blup | 70.0 | Dam 3 | 134534543 | -1.26 | 3.48 | 0.36 | 0.39 |
| 10 | Blup | 70.0 | Dam 4 | 134633225 | -0.93 | 3.87 | 0.24 | 0.43 |
| 10 | Blup | 70.0 | Dam 5 | 134567133 | 10.41 | 4.06 | 2.56 | **0.12** |
| 10 | Blup | 70.0 | Dam 6 | 134626172 | -8.54 | 3.58 | 2.39 | 0.13 |
| 10 | Blup | 70.0 | Dam 7 | 134561470 | -10.45 | 4.31 | 2.42 | **0.12** |
| 10 | Ebv | 72.0 | Dam 1 | 133565797 | 1.84 | 2.71 | 0.68 | 0.31 |
| 10 | Ebv | 72.0 | Dam 2 | 134919497 | -2.45 | 1.81 | 1.36 | 0.20 |
| 10 | Ebv | 72.0 | Dam 3 | 134534543 | -0.20 | 1.74 | 0.12 | 0.46 |
| 10 | Ebv | 72.0 | Dam 4 | 134633225 | -4.15 | 2.01 | 2.07 | 0.14 |
| 10 | Ebv | 72.0 | Dam 5 | 134567133 | 4.87 | 2.13 | 2.29 | 0.13 |
| 10 | Ebv | 72.0 | Dam 6 | 134626172 | -3.21 | 1.80 | 1.78 | 0.16 |
| 10 | Ebv | 72.0 | Dam 7* | 134561470 | **-7.18** | 2.36 | 3.04 | **0.10** |
| 12 | Ebv | 36.0 | Dam 1 | 133565797 | -0.64 | 2.70 | 0.24 | 0.43 |
| 12 | Ebv | 36.0 | Dam 2 | 134919497 | 0.52 | 4.46 | 0.12 | 0.46 |
| 12 | Ebv | 36.0 | Dam 3 | 134534543 | 1.88 | 2.52 | 0.75 | 0.30 |
| 12 | Ebv | 36.0 | Dam 4 | 134633225 | -5.71 | 2.08 | 2.74 | **0.11** |
| 12 | Ebv | 36.0 | Dam 5 | 134567133 | -0.22 | 2.15 | 0.10 | 0.47 |
| 12 | Ebv | 36.0 | Dam 6 | 134626172 | -5.69 | 1.91 | 2.97 | **0.10** |
| 12 | Ebv | 60.0 | Dam 7 | 134561470 | 4.11 | 2.18 | 1.89 | 0.16 |
| 12 | Ebv | 60.0 | Sire 1 | 134927390 | 2.64 | 2.51 | 1.05 | 0.24 |
| 12 | Ebv | 60.0 | Sire 2 | 134728092 | -2.86 | 1.39 | 2.05 | 0.14 |
| 12 | Ebv | 60.0 | Sire 3 | 134528597 | -0.72 | 1.33 | 0.54 | 0.34 |
| 12 | Ebv | 60.0 | Sire 4* | 134668746 | **-6.41** | 1.79 | 3.58 | **0.09** |
| 12 | Ebv | 60.0 | Sire 5 | 134914663 | 3.13 | 2.08 | 1.51 | 0.19 |
| 14 | Ebv | 95.0 | Sire 1 | 134927390 | 3.97 | 2.63 | 1.51 | 0.19 |
| 14 | Ebv | 95.0 | Sire 2 | 134728092 | 0.50 | 1.50 | 0.33 | 0.40 |
| 14 | Ebv | 95.0 | Sire 3 | 134528597 | 3.31 | 1.74 | 1.90 | 0.15 |
| 14 | Ebv | 95.0 | Sire 4* | 134668746 | **-6.00** | 2.32 | 2.59 | **0.12** |
| 14 | Ebv | 95.0 | Sire 5 | 134914663 | 4.59 | 2.42 | 1.90 | 0.15 |
| 16 | Blup | 45 | Sire 1 | 134927390 | -1.58 | 8.10 | 0.20 | 0.44 |
| 16 | Blup | 45 | Sire 2 | 134728092 | 6.89 | 4.26 | 1.62 | 0.18 |
| 16 | Blup | 45 | Sire 3 | 134528597 | -9.65 | 4.70 | 2.05 | 0.14 |
| 16 | Blup | 45 | Sire 4 | 134668746 | 13.29 | 6.21 | 2.14 | 0.14 |
| 16 | Blup | 45 | Sire 5* | 134914663 | **13.62** | 5.32 | 2.56 | **0.12** |
| 16 | Blup | 71 | Sire 1 | 134927390 | -6.18 | 7.90 | 0.78 | 0.29 |
| 16 | Blup | 71 | Sire 2 | 134728092 | -6.00 | 4.45 | 1.35 | 0.20 |
| 16 | Blup | 71 | Sire 3 | 134528597 | **16.99** | 5.06 | 3.36 | **0.09** |
| 16 | Blup | 71 | Sire 4 | 134668746 | -6.44 | 6.50 | 0.99 | 0.25 |
| 16 | Blup | 71 | Sire 5 | 134914663 | **-17.86** | 5.93 | 3.01 | **0.10** |
| 16 | Blup | 65.0 | Dam 1 | 133565797 | -3.81 | 4.56 | 0.84 | 0.28 |
| 16 | Blup | 65.0 | Dam 2 | 134919497 | -5.52 | 3.83 | 1.44 | 0.19 |
| 16 | Blup | 65.0 | Dam 3 | 134534543 | -7.70 | 3.39 | 2.27 | 0.13 |
| 16 | Blup | 65.0 | Dam 4 | 134633225 | 5.48 | 3.53 | 1.55 | 0.18 |
| 16 | Blup | 65.0 | Dam 5* | 134567133 | **-14.48** | 5.54 | 2.61 | **0.12** |
| 16 | Blup | 65.0 | Dam 6 | 134626172 | 7.23 | 6.68 | 1.08 | 0.24 |
| 16 | Blup | 65.0 | Dam 7 | 134561470 | -7.43 | 4.38 | 1.70 | 0.17 |
| 19 | Ebv | 40.0 | Sire 1 | 134927390 | 1.17 | 2.85 | 0.41 | 0.38 |
| 19 | Ebv | 40.0 | Sire 2 | 134728092 | 1.75 | 1.45 | 1.21 | 0.22 |
| 19 | Ebv | 40.0 | Sire 3* | 134528597 | **5.77** | 1.61 | 3.58 | **0.09** |
| 19 | Ebv | 40.0 | Sire 4 | 134668746 | -1.19 | 2.99 | 0.40 | 0.38 |
| 19 | Ebv | 40.0 | Sire 5 | 134914663 | 0.67 | 2.32 | 0.29 | 0.41 |
| 19 | Blup | 45.0 | Sire 1 | 134927390 | 3.15 | 4.93 | 0.64 | 0.32 |
| 19 | Blup | 45.0 | Sire 2 | 134728092 | 0.75 | 2.53 | 0.30 | 0.41 |
| 19 | Blup | 45.0 | Sire 3* | 134528597 | **9.58** | 2.60 | 3.69 | **0.08** |
| 19 | Blup | 45.0 | Sire 4 | 134668746 | 4.82 | 5.96 | 0.81 | 0.28 |
| 19 | Blup | 45.0 | Sire 5 | 134914663 | 1.90 | 3.83 | 0.50 | 0.35 |
| 19 | Blup | 64.0 | Dam 1 | 133565797 | -5.76 | 5.26 | 1.10 | 0.24 |
| 19 | Blup | 64.0 | Dam 2 | 134919497 | -7.11 | 4.79 | 1.49 | 0.19 |
| 19 | Blup | 64.0 | Dam 3* | 134534543 | **8.81** | 3.52 | 2.50 | **0.12** |
| 19 | Blup | 64.0 | Dam 4 | 134633225 | -2.11 | 5.16 | 0.41 | 0.38 |
| 19 | Blup | 64.0 | Dam 5 | 134567133 | 8.26 | 4.22 | 1.96 | 0.15 |
| 19 | Blup | 64.0 | Dam 6 | 134626172 | 7.88 | 3.45 | 2.28 | 0.13 |
| 19 | Blup | 64.0 | Dam 7 | 134561470 | 0.89 | 4.05 | 0.22 | 0.43 |
| 22 | Ebv | 35.0 | Dam 1 | 133565797 | 2.19 | 2.65 | 0.82 | 0.28 |
| 22 | Ebv | 35.0 | Dam 2 | 134919497 | 0.56 | 1.94 | 0.29 | 0.41 |
| 22 | Ebv | 35.0 | Dam 3 | 134534543 | -1.91 | 1.76 | 1.09 | 0.24 |
| 22 | Ebv | 35.0 | Dam 4* | 134633225 | **6.68** | 1.93 | 3.46 | **0.09** |
| 22 | Ebv | 35.0 | Dam 5 | 134567133 | -3.12 | 2.30 | 1.36 | 0.20 |
| 22 | Ebv | 35.0 | Dam 6 | 134626172 | -0.98 | 2.11 | 0.46 | 0.36 |
| 22 | Ebv | 35.0 | Dam 7 | 134561470 | 4.98 | 2.39 | 2.09 | 0.14 |
| 29 | Ebv | 50.0 | Sire 1 | 134927390 | 1250.98 | 1338.42 | 0.93 | 0.26 |
| 29 | Ebv | 50.0 | Sire 2 | 134728092 | 7.11 | 33.17 | 0.21 | 0.43 |
| 29 | Ebv | 50.0 | Sire 3* | 134528597 | **11951.38** | 4494.29 | 2.66 | **0.11** |
| 29 | Ebv | 50.0 | Sire 4 | 134668746 | 1014.84 | 426.57 | 2.38 | 0.13 |
| 29 | Ebv | 50.0 | Sire 5 | 134914663 | -117.09 | 94.42 | 1.24 | 0.22 |
| 29 | Ebv | 56.0 | Sire 1 | 134927390 | -1249.44 | 1338.02 | 0.93 | 0.26 |
| 29 | Ebv | 56.0 | Sire 2 | 134728092 | -7.21 | 33.05 | 0.22 | 0.43 |
| 29 | Ebv | 56.0 | Sire 3* | 134528597 | **-11961.37** | 4496.70 | 2.66 | **0.11** |
| 29 | Ebv | 56.0 | Sire 4 | 134668746 | -1010.67 | 426.38 | 2.37 | 0.13 |
| 29 | Ebv | 56.0 | Sire 5 | 134914663 | 112.76 | 94.79 | 1.19 | 0.22 |

^1^Half-sib regression interval mapping was performed with software GridQTL [[1](#_ENREF_1)].

^2^Rainbow trout chromosome number (OMY) is according to genetic maps from Rexroad *et al*. [[2](#_ENREF_2)].

^3^Stress response traits: (1) Animal estimated breeding value (EBV) using four repeated measurements of plasma cortisol was calculated with software iBay version 1.46 [[3](#_ENREF_3)]; and (2) Index best least unbiased predictor (BLUP) from three repeated measurements weighted by their relative heritability was estimated with software ASReml version 2.0 [[4](#_ENREF_4)].

^4^QTL location from HS regression interval mapping performed with software GridQTL [[1](#_ENREF_1)].

^5^Within each trait QTL group, the sire or dam parent that is most likely segregating the QTL allele is indicated with an asterisk (*), and the parent with the lowest *P*-value is bold text highlighted. The effect of QTL with suggestive/significant effect detected in this study is yellow highlighted.

^6^The QTL effect is expressed in ng/mL of plasma cortisol.

^7^The allele substitution effect for each parent was tested using a one-sided t-test (testing absolute *t*-values) with one DF.

**References**

1. Seaton G, Hernandez J, Grunchec JA, White I, Allen J, De Koning DJ, Wei W, Berry D, Haley C, Knott S: **GridQTL: A Grid Portal for QTL Mapping of Compute Intensive Datasets**. In: *8th World Congress on Genetics Applied to Livestock Production: August 13-18, 2006 2006; Belo Horizonte, Brazil*; 2006.

2. Rexroad CE, 3rd, Palti Y, Gahr SA, Vallejo RL: **A second generation genetic map for rainbow trout (Oncorhynchus mykiss)**. *BMC Genet* 2008, **9**:74.

3. Janss LLG: **iBay manual version 1.46**. Leiden, Netherlands: Janss Biostatistics, P.O. Box 535; 2008.

4. Gilmour AR, Gogel, B.J., Cullis, B.R., and Thompson, R.: **ASReml User Guide Release 2.0**. Hemel Hempstead, HP1 1ES, UK: VSN International Ltd; 2006.
